# Dual Delivery of BMP-2 and bFGF from a New Nano-Composite Scaffold, Loaded with Vascular Stents for Large-Size Mandibular Defect Regeneration

**DOI:** 10.3390/ijms140612714

**Published:** 2013-06-18

**Authors:** Jiansheng Su, Hongzhen Xu, Jun Sun, Xue Gong, Hang Zhao

**Affiliations:** Institute of Prosthodontics, School of Stomatology, Tongji University, 399 Yanchang Road, Shanghai 200092, China

**Keywords:** bone morphogenetic protein-2, basic fibroblast growth factor, tissue engineering, bone defect, poly(lactic-*co*-glycolic acid), polycaprolactone, hydroxyapatite

## Abstract

The aim of this study was to investigate the feasibility and advantages of the dual delivery of bone morphogenetic protein-2 (BMP-2) and basic fibroblast growth factor (bFGF) from nano-composite scaffolds (PLGA/PCL/nHA) loaded with vascular stents (PLCL/Col/nHA) for large bone defect regeneration in rabbit mandibles. Thirty-six large bone defects were repaired in rabbits using engineering bone composed of allogeneic bone marrow mesenchymal stem cells (BMSCs), bFGF, BMP-2 and scaffolds composed of PLGA/PCL/nHA loaded with PLCL/Col/nHA. The experiments were divided into six groups: BMSCs/bFGF/BMP-2/scaffold, BMSCs/BMP-2/scaffold, BMSCs/bFGF/scaffold, BMSCs/scaffold, scaffold alone and no treatment. Sodium alginate hydrogel was used as the carrier for BMP-2 and bFGF and its features, including gelling, degradation and controlled release properties, was detected by the determination of gelation and degradation time coupled with a controlled release study of bovine serum albumin (BSA). AlamarBlue assay and alkaline phosphatase (ALP) activity were used to evaluate the proliferation and osteogenic differentiation of BMSCs in different groups. X-ray and histological examinations of the samples were performed after 4 and 12 weeks post-implantation to clarify new bone formation in the mandible defects. The results verified that the use of sodium alginate hydrogel as a controlled release carrier has good sustained release ability, and the combined application of bFGF and BMP-2 could significantly promote the proliferation and osteogenic differentiation of BMSCs (*p* < 0.05 or *p* < 0.01). In addition, X-ray and histological examinations of the samples exhibited that the dual release group had significantly higher bone formation than the other groups. The above results indicate that the delivery of both growth factors could enhance new bone formation and vascularization compared with delivery of BMP-2 or bFGF alone, and may supply a promising way of repairing large bone defects in bone tissue engineering.

## 1. Introduction

Large bone defects of the oral and maxillofacial regions caused by trauma, inflammation, cancer and congenital malformations have been a significant problem in stomatology [1]. Traditional techniques for repairing bone defects mainly include the implantation of bone autografts, allografts, xenografts and artificial bones [2,3]. However, autologous bone grafts are considered to be the gold standard for repairing and reconstructing bone defects as they have good osteoconductive and osteoinductive properties, their application is hampered due to the limited donor tissue and secondary damage [4]. Xenografts and artificial bone implants also have many disadvantages, including poor biocompatibility, and increased risk of infection and/or rejection [5]. Bone tissue engineering has been considered as the most promising solution for repairing bone defects in clinical practice, which may help to optimize an efficient induction of bone formation via the delivery of multiple morphogenetic signaling factors [6–8]. During the process of bone regeneration, cell growth factors, such as BMPs, bFGF, and VEGF, regulate cellular behavior and ultimately the tissue response. The strong spatial and temporal control under which these growth factors are presented to cells under normal conditions, stresses the relevance of spatiotemporally controlling the therapeutic delivery of multiple factors for bone tissue regeneration. BMP is a polypeptide growth factor derived from demineralized bone matrix, which was originally named after its role in the induction of bone formation. BMPs are a group of secreted, hydrophobic, acid glycoproteins that can induce the differentiation of osteoblasts *in vitro*. So far at least forty members were founded in the family of BMPs, which have different biological effects [6,9,10]. BMP-2 is one of the most important members of the BMP family and is widely considered to be the strongest factor for osteogenic induction. Thus, BMP-2 is considered the most promising factor for bone tissue engineering and is an essential component of the signaling pathway controlling fracture repair [11]. As a member of the fibroblast growth factor family, FGF is synthesized in various organs and cells, and is a powerful mitogenic factor, with a variety of biological activities, including the promotion of cell proliferation and differentiation. FGF can be divided into two types, one is acidic fibroblast growth factor (aFGF), and the other is basic fibroblast growth factor (bFGF). bFGF can increase cellular content of osteocalcin and the number of osteoblasts by promoting DNA synthesis and mitosis of bone cells [7,12]. Several studies have shown that bFGF is stronger than aFGF in bone reconstruction [13–15]. The process of bone formation starts by the induction of BMPs, which is subject to comprehensive regulation by several growth factors. This synergy will be achieved by using the correct combination of growth factors to promote better bone regeneration and vascularization [16]. Enhanced vascularization and bone regeneration has been reported for combined treatment of BMP-2 and bFGF in comparison to the single use of either BMP-2 or bFGF in many previous literatures [17–19].

Scaffold materials are one of the important components in bone tissue engineering. The optimal scaffold should be three-dimensional and contain a highly porous network of interconnected pores to promote cell growth, adhesion, proliferation and differentiation by facilitating the flow and transport of nutrients and metabolic waste. In addition, they should have good biocompatibility and biodegradability to assure they are non-toxic, non-allergenic and do not induce any adverse reactions in the body. The formation of new tissues should also match the degradation rate of the implanted materials to prevent further damage. Finally, good mechanical properties are also the essential requirements of an ideal scaffold in bone tissue engineering [20–23]. In this study, we used nano-composite materials of PLGA/PCL/nHA loaded with vascular stents of PLCL/Col/nHA as the carrier for BMSCs, which have been confirmed in our previous studies to meet the basic requirements of an ideal scaffold for possessing good biocompatibility, biodegradability, biological activity, porosity and mechanical properties [24].

Currently, there are many methods of achieving a sustained-release of growth factors, including the use of ceramics and non-hydrated polymers [25,26]; however, hydrogels are undoubtedly the better candidate as they are generally biocompatible, biodegradable, and in most cases, injectable [27–33]. Sodium alginate is a polysaccharide carbohydrate extracted from the brown algae of seaweed or Sargasso, which is composed of 1,4-poly-13-d-mannose, glucuronic acid and a-l-guluronic uronic acid [34]. The formula of sodium alginate is (C_6_H_7_O_6_Na)*_n_* and the relative molecular weight is about 32,000 to 200,000, with 198.11 being the structural unit molecular weight of the theoretical value. Sodium alginate can form a gel in the presence of divalent ions, such as Ca^2+^ and Ba^2+^, with the gel formed with calcium chloride showing the maximum strength. Nowadays, sodium alginate is widely used in biological engineering, tissue engineering, and medicine as it has good biocompatibility, a mild gelling process and good performance in the controlled release of factors [35,36]. In this study, the nano-composite scaffolds and hydrogel which was formed with sodium alginate and calcium chloride, were applied as the growth matrix for BMSCs and the sustained-release carrier for the growth factors, BMP-2 and bFGF, which were expected to generate better effectiveness for osteogenesis and vascularization.

## 2. Results and Discussion

### 2.1. Properties of Sodium Alginate Hydrogel

**Figure 1:**
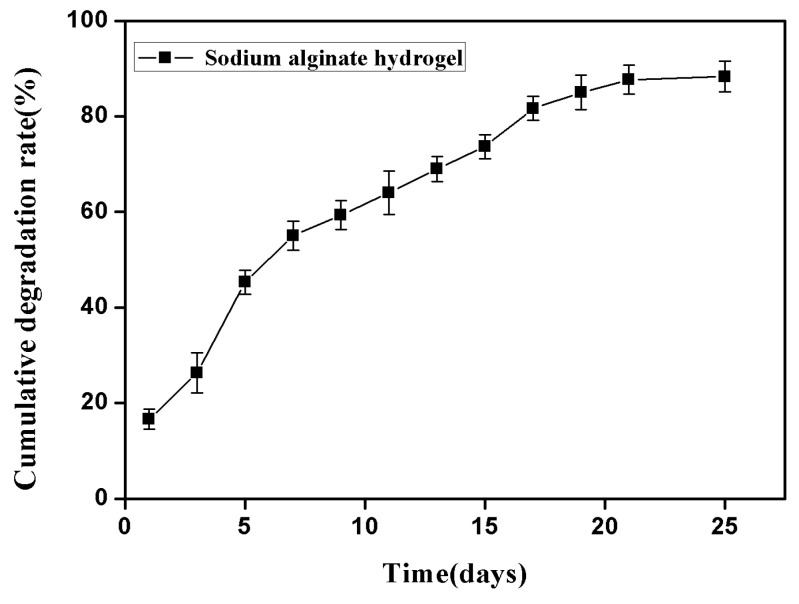
*In vitro* degradation of sodium alginate hydrogel formed with 3% sodium alginate aqueous solution and 5% aqueous calcium chloride solution in phosphate buffer solution (PBS) for 25 days at 37 °C.

The hydrogel prepared with a 3% (*w*/*v*) aqueous solution of sodium alginate and 5% (*w*/*v*) aqueous solution of anhydrous calcium chloride showed a fast and ideal gelation time of 12–17 s, which was colorless and transparent, and had the characteristics of colloidal thickness, good elasticity and high tensile strength. As shown in Figure 1, the degradation experimental results of the sodium alginate hydrogel *in vitro* showed that it had a faster degradation in the first week, reaching 54.67%. It then entered a relatively slow degradation period during the second week, with a total degradation rate of 72%. In the third week, it still showed a relatively slow degradation rate, but after 21 days, most of the hydrogel had been completely degraded, with the ultimate degradation rate reaching almost 90%.

Bovine serum albumin (BSA) is often used as a model protein in the analysis of sustained-release property of various carriers, such as nano-capsules, microspheres, and hydrogels, as it has similar performance as growth factors in these studies and is less costly [37–39].

**Figure 2:**
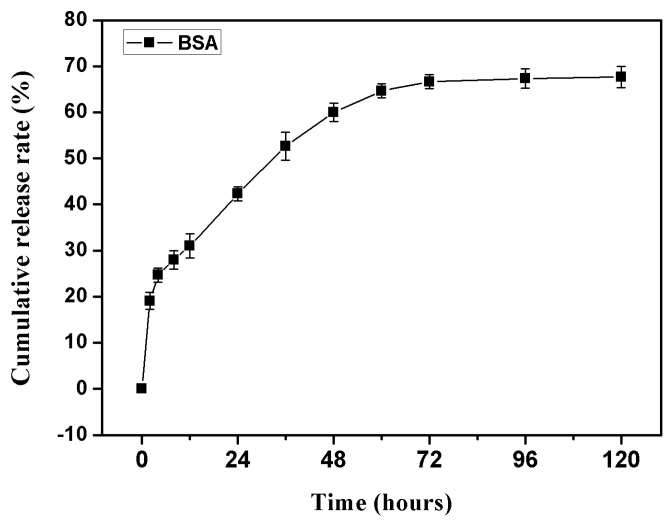
Cumulative release rate of bovine serum albumin (BSA) per time point from sodium alginate hydrogel.

In this study, BSA was used as a model protein instead of growth factors in a preliminary study of the controlled release performance of the sodium alginate hydrogel *in vitro*. The results, as shown in Figure 2, demonstrate that BSA-hydrogel had a specific release profile, where, although there was a 19.1% burst release within the first two hours and the cumulative release rate reached 42.33% in the first day, there was a slow and uniform release process in the subsequent seventy-two hours. The preliminary experimental study on the controlled release of BSA lasted one hundred twenty hours, in which the cumulative release rate tended to balance out after a period of slow growth and ultimately reached 67.67%.

### 2.2. Bioactivity and Biocompatibility *in Vitro*

**Figure 3:**
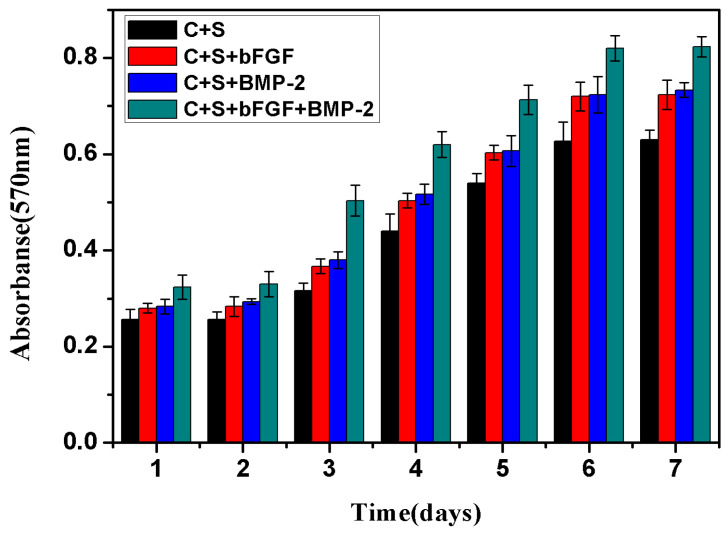
Four groups of cell proliferation were analyzed by alamarBlue assay: C + S, BMSCs/scaffold; C + S + bFGF, BMSCs/bFGF/scaffold; C + S + BMP-2, BMSCs/BMP-2/scaffold; C + S + bFGF + BMP-2, BMSCs/bFGF/BMP-2/scaffold.

The BMSCs were cultured in different groups, including BMSCs/scaffold, BMSCs/bFGF/scaffold, BMSCs/BMP-2/scaffold, BMSCs/bFGF/BMP-2/scaffold. The cell proliferation in each group was evaluated using the alamarBlue assay. As shown in Figure 3, during 7 days of culture, the number of living cells increased in all four groups, and showed significant growth in the BMSCs/bFGF/BMP-2/scaffold group compared with the other three groups (*p* < 0.05 or *p* < 0.01). The cell proliferation in the BMSCs/BMP-2/scaffold and BMSCs/bFGF/scaffold groups appeared to have similar behavior and were significantly faster than the BMSCs/scaffold group (*p* < 0.05). These results indicate that both growth factors, BMP-2 and bFGF, could promote the proliferation of BMSCs and the combined application was better than using either alone.

Wang Lei *et al.* had confirmed that both BMP-2 (100 ng/mL) and bFGF (50 ng/mL) were good at promoting BMSC proliferation in their previous studies, and the use of BMP-2 and bFGF at a ratio of 2:1 (100:50 ng/mL) could significantly promote the proliferation and differentiation of BMSCs compared with BMP-2 or bFGF alone [40]. The optimal concentration of BMP-2 and bFGF in Wang Lei *et al* studies was used in our research and the obtained results were consistent with their previous findings.

**Figure 4:**
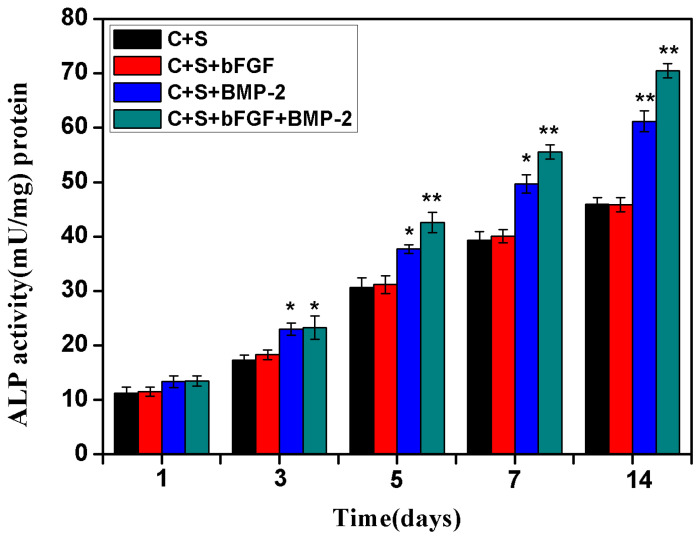
Changes in alkaline phosphatase (ALP) activity of BMSCs in all four groups on culture days 1, 3, 5, 7 and 14. * A value of *p* < 0.05 and ** A value of *p* < 0.01 were considered statistically significant.

ALP activity was assessed on BMSCs seeded on the scaffolds on culture days 1, 3, 5, 7 and 14 in all four groups (Figure 4). At day 1, the ALP activity of the four groups of BMSCs was low and there was no significant difference, indicating there was little induction of stem cells to osteogenic differentiation in the initial culture day. From day 3, the differences of ALP activity between the four groups began to appear. The level of ALP activity in the BMSCs/bFGF/BMP-2/scaffold and BMSCs/BMP-2/scaffold groups was higher than the other two groups (*p* < 0.05). As the culture period progressed, although the ALP activity levels increased in all groups, it showed more rapid growth in the BMSCs/bFGF/BMP-2/scaffold and BMSCs/BMP-2/scaffold groups compared with the other two groups (*p* < 0.05 or *p* < 0.01), indicating that both the combined application of BMP-2 and bFGF and individual application of BMP-2 could induce BMSCs towards osteogenic differentiation. Between the BMSCs/bFGF/BMP-2/scaffold and BMSCs/BMP-2/scaffold groups, the former had higher ALP activity, indicating that the combined application of BMP-2 and bFGF had a greater osteogenic induction effect than BMP-2 alone. In addition, the BMSCs/bFGF/scaffold group showed similar characteristics and trends in ALP activity to the BMSCs/scaffold group, which confirmed that bFGF alone cannot promote BMSC differentiation into osteoblasts.

### 2.3. *In Vitro* Release of bFGF and BMP-2 from Sodium Alginate Hydrogel and Nano-Composite Scaffolds (PLGA/PCL/nHA) Loaded with Vascular Stents (PLCL/Col/nHA)

**Figure 5:**
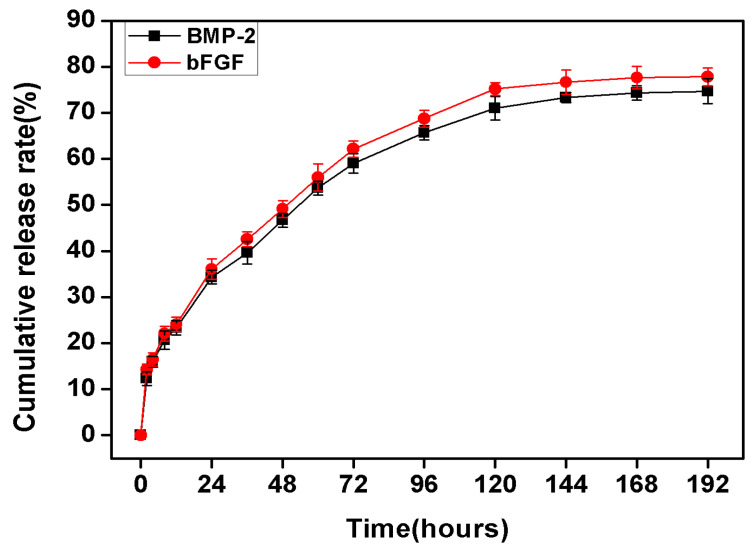
Cumulative release rate of bFGF and BMP-2 per time point from sodium alginate hydrogel and nano-composite scaffolds (PLGA/PCL/nHA) loaded with vascular stents (PLCL/Col/nHA).

The *in vitro* release kinetics of bFGF and BMP-2 in the BMSCs/bFGF/BMP-2/scaffold group was studied using ELISA quantitative detection kits for BMP-2 and bFGF. As shown in Figure 5, the *in vitro* release kinetics of both growth factors presented similar release properties. Firstly, a burst release of about 35% occurred for both growth factors within the first 24 hours, which is lower than the first day of release for BSA in the hydrogel. Over the next four days, a steady and slow release was maintained, and the release rate of growth factors per day was about 6% or 7%. From the fifth day, the release rates of both growth factors were significantly reduced to 2% or 1%. After 7 days, the release ceased and the ultimate cumulative release of bFGF and BMP-2 were 77.83% and 74.67%, respectively, and there was no statistically significant difference (*p* < 0.05). Compared with the results of a previous study of the release of BSA from the sodium alginate hydrogel, the release kinetics of the two growth factors from the BMSCs/bFGF/BMP-2/scaffold groups showed better performance in controlled-release, which was stable, slow and more prolonged. The double controlled-release carriers of sodium alginate hydrogel and nano-composite scaffolds (PLGA/PCL/nHA) loaded with vascular stents (PLCL/Col/nHA) may be the result of the above phenomenon as the porous nano-composite scaffolds themselves also have certain sustained release capabilities, which has been confirmed in previous studies [41,42].

Maintenance of growth factors to carrier materials is crucial to ensure prolonged therapeutic efficacy of growth factor delivery and avoid premature loss of growth factors. In our study, alginate hydrogel formed with 3% sodium alginate aqueous solution and 5% aqueous calcium chloride solution was used as the sustained-release carrier for the growth factors, BMP-2 and bFGF. Although the results of BSA-loaded release study (Figure 2) and BMP-2 or/and bFGF-loaded release test (Figure 5) showed long-term, stable, slow release phenomenon, nearly 35% of the burst release is still appear in the first twenty-four hours, which is consistent with previously reported literature [43,44]. Improving hydrogel performance and reducing the value of the burst release is still a difficult problem which needs to be addressed in future studies. Ideal hydrogel materials should consist of long-term, stable, slow release growth factors, and display remarkably low burst release values.

### 2.4. Histological Analysis

**Figure 6:**
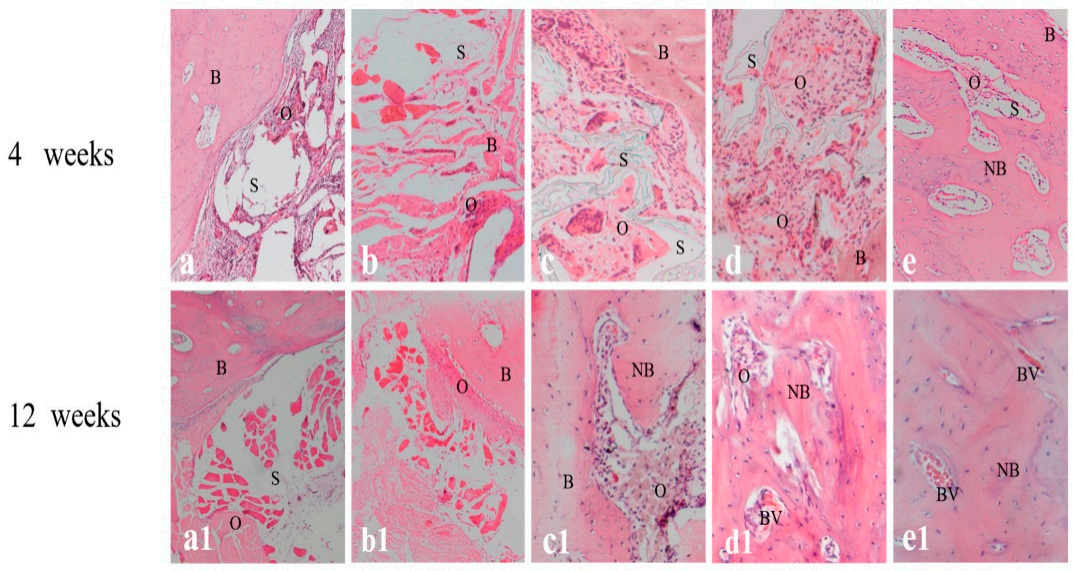
Tissue slices of different mandibular implants at weeks 4 and 12 after staining with hematoxylin and eosin (H&E). (**a**,**a1**) Scaffold alone; (**b**,**b1**) BMSCs/scaffold; (**c**,**c1**) BMSCs/bFGF/scaffold; (**d**,**d1**) BMSCs/BMP-2/scaffold; (**e**,**e1**) BMSCs/bFGF/BMP-2/scaffold. B, bone; S, scaffold; O, osteoid matrix; NB, new bone; BV, blood vessel.

New bone formation was found in the implanted area in all groups, except the no treatment group where no animals survived, thus demonstrating that rabbit mandibular 26 mm large-size bone defects cannot repair themselves without the use of implants, and ultimately results in death. The amount of new bone formation in the BMSCs/BMP-2/scaffold and BMSCs/bFGF/BMP-2/scaffold groups was significantly more than the other three groups, and compared with the BMSCs/BMP-2/scaffold group, the BMSCs/bFGF/BMP-2/scaffold group showed better osteogenic effects at weeks 4 and 12. As shown in Figure 6, osteoid matrix mainly occurred near the junction formed between the graft and normal bone in the scaffold alone group, whereas it also formed inside the stents by the bone wall in the BMSCs/scaffold and BMSCs/bFGF/scaffold groups, which had similar osteogenic performances. Large amounts of osteoid matrix formed in the inner space of the BMSCs/BMP-2/scaffold and BMSCs/bFGF/BMP-2/scaffold implants by week 4 post-implantation, which showed increased osteogenic effects compared with the other three groups, especially in the BMSCs/bFGF/BMP-2/scaffold group, where new bone similar to natural bone formed in the inner space of the stents. At week 12, we could see increased bone matrix in the scaffold alone and BMSCs/scaffold groups compared to the week 4 samples, but no significant new bone formation could be observed. However, in the BMSCs/bFGF/scaffold, BMSCs/BMP-2/scaffold and BMSCs/bFGF/BMP-2/scaffold groups significant new bone formation and integration at the boundary between the bone and implants could be observed, with the latter two groups having completely filled the bone defect area with new bone. Furthermore, as compared with the BMSCs/BMP-2/scaffold group, there was more mature new bone and blood vessel formation in the BMSCs/bFGF/BMP-2/scaffold group.

**Figure 7:**
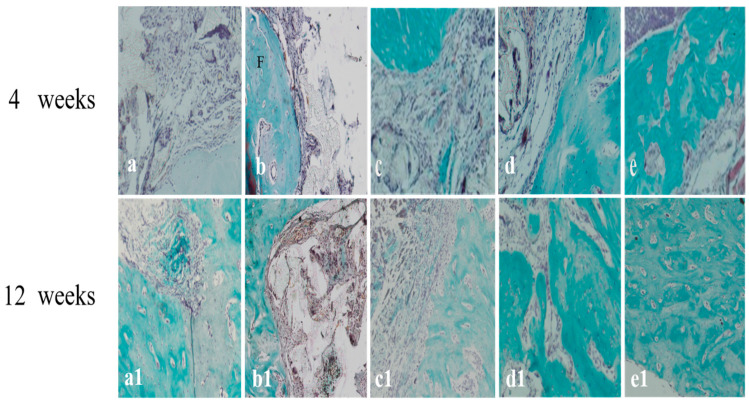
Tissue slices of different mandibular implants at weeks 4 and 12 of Masson staining. (**a**,**a1**) Scaffold alone; (**b**,**b1**) BMSCs/scaffold; (**c**,**c1**) BMSCs/bFGF/scaffold; (**d**,**d1**) BMSCs/BMP-2/scaffold; (**e**,**e1**) BMSCs/bFGF/BMP-2/scaffold; F, collagen fibers.

The tissue slices of different mandibular implants at 4 and 12 weeks were stained with Masson stain. The results, shown in Figure 7, revealed that fibrous collagens were secreted by BMSCs in all groups except the scaffold alone group at week 4, and the amount of fibrous collagen secretion occurring in the BMSCs/BMP-2/scaffold and BMSCs/bFGF/BMP-2/scaffold groups was significantly more than the BMSCs/scaffold and BMSCs/bFGF/scaffold groups. At week 12, small amounts of fibrous collagen expression was observed in the scaffold alone group, which could have been secreted from autologous bone marrow mesenchymal stem cells. From BMSCs/scaffold to BMSCs/bFGF/BMP-2/scaffold group, the expression of fibrous collagen gradually increased and in the BMSCs/BMP-2/scaffold and BMSCs/bFGF/BMP-2/scaffold group they were significantly more than in the BMSCs/scaffold and BMSCs/bFGF/scaffold groups.

These histological analysis results show that the dual release group had significantly higher new bone formation than the scaffold alone, BMSCs/scaffold and BMSCs/bFGF/scaffold groups. Furthermore, there was better promotion of bone and blood vessel formation in this group compared with the BMSCs/BMP-2/scaffold group.

### 2.5. Radiographic Observations

**Figure 8:**
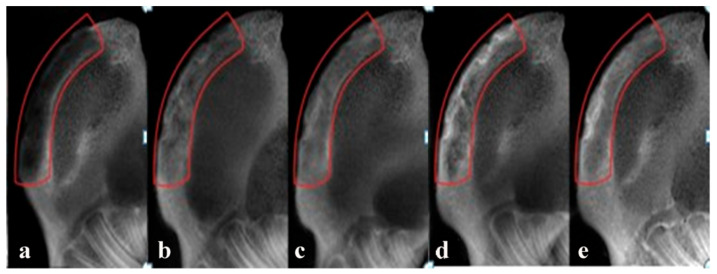
X-ray radiographs of large-sized mandibular defects repaired using different implants at week 12 post-implantation. (**a**) Scaffold alone; (**b**) BMSCs/scaffold; (**c**) BMSCs/bFGF/scaffold; (**d**) BMSCs/BMP-2/scaffold; (**e**) BMSCs/bFGF/BMP-2/scaffold.

At 12 weeks post-implantation, the implanted area of the alone group showed a low-density image and new bone formation only slightly increased in the edges of the defect areas (Figure 8a). The density of implanted areas markedly enhanced in the BMSCs/scaffold and BMSCs/bFGF/scaffold groups and there were no clear boundaries between the graft and the natural bone. Only a small amount of low-density shadow was visible in the middle of the defect area (Figure 8b,c). The radio density in the bone defects area of the BMSCs/BMP-2/scaffold and BMSCs/bFGF/BMP-2/scaffold groups was enclosed in the surrounding normal bone. In particular, in the BMSCs/bFGF/BMP-2/scaffold group, a uniform high-density image demonstrated excellent osteogenesis (Figure 8d,e).

**Figure 9:**
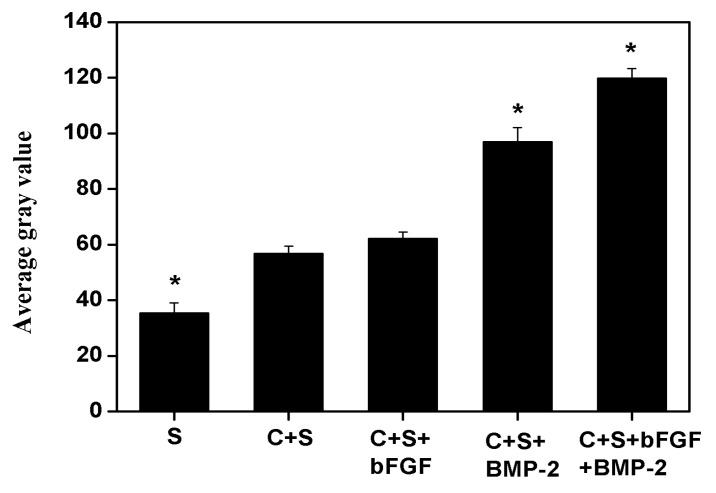
The analysis of new bone density of large-sized mandibular defects repaired using different implants shown at week 12 post-implantation. (Mean ± SD; *n* = 3; * *p* < 0.05).

The changes in the average grayscale value of bone mineral density of each group defect area clearly represented the level of new bone formation in each group (Figure 9). The dual delivery of BMP-2 and bFGF achieved significantly higher bone formation than the other experimental groups (*p* < 0.05). The application of BMP-2 alone also showed better osteogenic effects compared with the scaffold alone, BMSCs/scaffold and BMSCs/bFGF/scaffold groups, but was lower than the dual delivery group. The scaffold alone group showed the lowest bone formation, illustrating that bone repair was less effective without the involvement of BMSCs and/or growth factors.

At present, repair of large bone defects is a significant challenge for clinics, even though critical sized bone defects have been well repaired in many animal experiments. In our research, large sized bone defect models of rabbit mandibles (26 × 5 × 3 mm) were prepared which exceeded the critical size currently reported with a basic length of about 15 mm [45,46]. We achieved large-size mandibular defect regeneration via the dual delivery of BMP-2 and bFGF from a new nano-composite scaffold loaded with vascular stents and this may provide a new method for the repair of large bone defects.

## 3. Experimental Section

### 3.1. BMSC Culture and Seeding

New Zealand rabbit bone marrow mesenchymal stem cells (BMSCs) were isolated from the tibiae by a previously reported method of bone marrow washing combined with density gradient centrifugation [24]. Briefly, the isolated BMSCs were suspended in Dulbecco’s-modified Eagle medium (DMEM) with 10% fetal bovine serum (FBS) (Gibco BRL), 100 U/mL penicillin and 100 μg/mL streptomycin, and incubated at 37 °C with 5% humid CO_2_. The media was changed every 3 days, and when cells reached 80% confluence at 8–10 days, they were suspended for passage. BMSCs of passage 3 were used to seed on scaffolds and for *in vitro* and *in vivo* experiments.

### 3.2. Preparation and Performance Testing of Sodium Alginate Hydrogel

#### 3.2.1. Hydrogel Preparation

Sodium alginate (Sigma, St.Louis, MO, USA) and anhydrous calcium chloride powder (Sinopharm, Beijing, China) were sterilized for 30 min by high temperature and pressure (121 °C, 108 kPa), then dissolved in sterile double distilled water to prepare 1%, 3%, 5% (*w*/*v*) sodium alginate aqueous solutions and 1%, 3%, 5% (*w*/*v*) anhydrous calcium chloride solutions, respectively. Equal volumes of different concentrations of aqueous calcium chloride and sodium alginate aqueous solutions were mixed, fast shock oscillated and left standing to test the gelation time of the hydrogels. The ideal sodium alginate hydrogel was selected according to suitable gelling time and degradation rate. Briefly, the sodium alginate hydrogel formed with 1 mL 3% sodium alginate aqueous solution and 1 mL 5% aqueous calcium chloride solution in 5 mL centrifuge tubes with round-bottomed lids was placed in 3 mL phosphate buffer solution (PBS) and incubated at 37 °C. The degradation rate of sodium alginate hydrogel was detected by measuring the dry weight of hydrogel at different time points, with at least four samples being tested per time point to prepare a degradation curve.

#### 3.2.2. Protein-Loaded Release Study

Bovine serum albumin (BSA) (20 mg/mL) and 3% (*w*/*v*) sodium alginate solution was uniformly mixed together, and an equal volume of 5% (*w*/*v*) aqueous calcium chloride solution was added to the mixture, then waited for the mixture into gel. The BSA-containing gel blocks were placed in 20 mL PBS buffer solution (pH 7.4) at room temperature, and the BSA content of the released fluid was measured regularly using a UV spectrophotometer at 280 nm.

### 3.3. Fabrication of BMP-2 and bFGF-Loaded Scaffold

BMSCs of passage 3 (1 × 10^7^ cells/implant) were seeded on PLGA/PCL/nHA loaded with vascular stents (26 × 5 × 3 mm^3^), and after three days the composites of cells and scaffolds were soaked in the sodium alginate solution containing the two growth factors for 10 min, then removed and placed in anhydrous calcium chloride aqueous solution for 10 min. The implants including BMSCs/scaffold, BMSCs/bFGF/scaffold, BMSCs/BMP-2/scaffold and BMSCs/bFGF/BMP-2/scaffold were prepared and subjected to alamarBlue assay and alkaline phosphatase (ALP) activity tests to evaluate the proliferation of BMSCs and ALP activity on culture days 1, 3, 5, 7 and 14. Briefly, the proliferation of BMSCs was detected using alamarBlue assay kit purchased from Biosource International Company (Camarillo, CA, USA), and the experimental procedure was performed in accordance with the kit instructions. In addition, cells on implants were digested with trypsin and rinsed three times with PBS. Cells were pelleted by centrifugation and re-suspended in 100 μL PBS, following which they were freeze-thawed three times to disrupt the cell membrane. ALP activity assay kit (Wako Chemicals, Richmond, VA, USA) was used to test ALP activity, which measured the 405 nm absorbance value using p-nitrophenyl phosphate as the substrate. Total protein content was determined using Coomassie brilliant blue as described elsewhere [47]. The ALP activity was expressed as unit per mg protein. In addition, the *in vitro* release kinetics of the two growth factors in the BMSCs/bFGF/BMP-2/scaffold group were measured using BMP-2 and bFGF ELISA quantitative detection kit (R & D System, Emeryville, CA, USA), with strict adherence to the kit instructions.

### 3.4. Creation and Reconstruction of Large Bone Defect

Thirty-six healthy New Zealand white rabbits, weighing approximately 2.5 kg, were divided into six groups: scaffold alone (*n* = 6); BMSCs/scaffold (*n* = 6); BMSCs/bFGF/scaffold (*n* = 6); BMSCs/BMP-2/scaffold (*n* = 6); BMSCs/bFGF/BMP-2/scaffold (*n* = 6); no treatment (*n* = 6). The rabbits were anesthetized with pentobarbital sodium (0.2 mL/kg) and a 3 cm incision was made in the bilateral jaw. A large-size defect of 26 × 5 × 3 mm^3^ was made in a buccolingual direction on both sides of the mandible. The same size porous scaffolds or scaffold/cell/growth factors composites were inserted into the defects. Three rabbits randomly selected from each group were sacrificed at 4 and 12 weeks after implantation. The implants, together with surrounding tissue, were excised and the specimen tissue blocks were harvested and processed for X-ray detection and histological evaluation.

#### 3.4.1. Radiographic Observations

The experimental means and methods have been reported in our prior research [24]. Briefly, after sacrifice, lateral radiographs of the mandibles were taken with the mandibles positioned at 10 cm from the X-ray tube. The X-ray unit (Gendex DEN S-O-MAT, Milano, IL, USA) was set at 70 kV and 7 mA with a 0.26 s exposure time. The changes in average gray value in the bone defect areas were analyzed using the Jetta pathological image analysis software (Jetta Science and Technology Development Co., Ltd., Nanjing, China).

#### 3.4.2. Histological Analysis

After fixation, decalcification, dehydration, paraffin wax embedding, the tissue block specimens were cut into 5-μm-thickness sections. Following this, hematoxylin and eosin or Masson staining were employed to test new bone formation and expression of fibrous collagen, respectively, within and surrounding the implant. Slices were observed through a light microscope.

### 3.5. Statistical Analysis

All data were expressed as means ± SD (standard deviation). One-way analysis of variance (ANOVA) and Student’s *t* test were conducted to compare differences between groups using SPSS 14.0. Differences were considered to be significant when *p* < 0.05 or *p* < 0.01.

## 4. Conclusions

Nano-composite scaffolds (PLGA/PCL/nHA) loaded with vascular stents (PLCL/Col/nHA), BMSCs, BMP-2, and bFGF were combined to construct five different implants for large-size mandibular defect regeneration as follows: scaffold alone; BMSCs/scaffold; BMSCs/bFGF/scaffold; BMSCs/BMP-2/scaffold; BMSCs/bFGF/BMP-2/scaffold. The scaffold containing the controlled-release carriers for BMP-2 and bFGF were confirmed to have good sustained release properties. The *in vitro* and *in vivo* bioactivity and osteogenesis abilities of the composite implants were evaluated using ALP activity tests, histological analysis and radiographic observations. The results of this study demonstrate that the BMSCs/bFGF/BMP-2/scaffold composite implants showed increased bone and vascular formation, compared with the composite implant containing a single growth factor, and were capable of repairing large-sized mandibular defects in New Zealand white rabbit models. This composite may be an alternative and promising repair material for bone defects.
