# Correction: Su et al. Dual Delivery of BMP-2 and bFGF from a New Nano-Composite Scaffold, Loaded with Vascular Stents for Large-Size Mandibular Defect Regeneration. *Int. J. Mol. Sci.* 2013, *14*, 12714–12728

**DOI:** 10.3390/ijms26178730

**Published:** 2025-09-08

**Authors:** Jiansheng Su, Hongzhen Xu, Jun Sun, Xue Gong, Hang Zhao

**Affiliations:** Institute of Prosthodontics, School of Stomatology, Tongji University, 399 Yanchang Road, Shanghai 200092, China; 021xuhongzhen@tongji.edu.cn (H.X.); cipher44444444@hotmail.com (J.S.); gongxue0727@163.com (X.G.); 2011zhaohang@tongji.edu.cn (H.Z.)

In the original publication [1], there were mistakes in Figures 6 and 7 as published. The mistakes were made due to uploading incorrect images and not carefully checking them back then. The corrected Figure 6 and Figure 7 appear below. The authors state that the scientific conclusions are unaffected. This correction was approved by the Academic Editor. The original publication has also been updated.

## Figures and Tables

**Figure 6:**
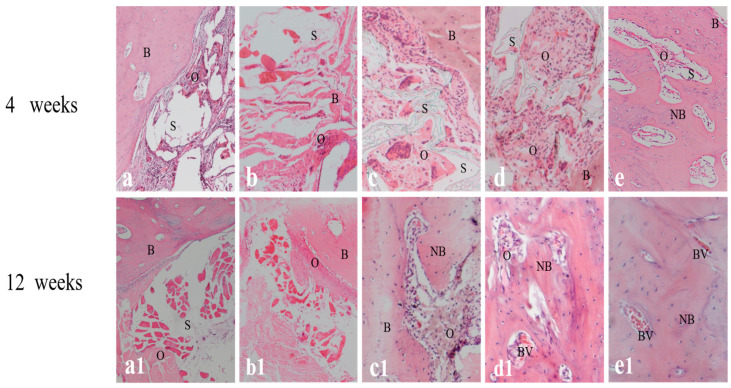
Tissue slices of different mandibular implants at weeks 4 and 12 after staining with hematoxylin and eosin (H&E). (**a**,**a1**) Scaffold alone; (**b**,**b1**) BMSCs/scaffold; (**c**,**c1**) BMSCs/bFGF/scaffold; (**d**,**d1**) BMSCs/BMP-2/scaffold; (**e**,**e1**) BMSCs/bFGF/BMP-2/scaffold. B, bone; S, scaffold; O, osteoid matrix; NB, new bone; BV, blood vessel.

**Figure 7:**
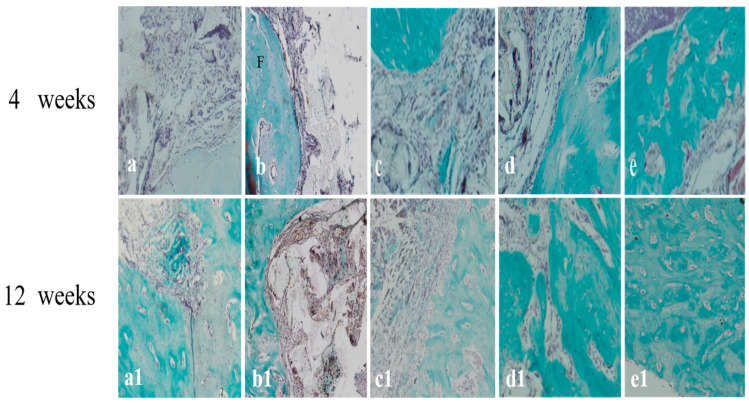
Tissue slices of different mandibular implants at weeks 4 and 12 of Masson staining. (**a**,**a1**) Scaffold alone; (**b**,**b1**) BMSCs/scaffold; (**c**,**c1**) BMSCs/bFGF/scaffold; (**d**,**d1**) BMSCs/BMP-2/scaffold; (**e**,**e1**) BMSCs/bFGF/BMP-2/scaffold; F, collagen fibers.

## References

[1] Su J., Xu H., Sun J., Gong X., Zhao H. (2013). Dual Delivery of BMP-2 and bFGF from a New Nano-Composite Scaffold, Loaded with Vascular Stents for Large-Size Mandibular Defect Regeneration. Int. J. Mol. Sci..

